# Nitrotriazole-based acetamides and propanamides with broad spectrum antitrypanosomal activity

**DOI:** 10.1016/j.ejmech.2016.08.002

**Published:** 2016-11-10

**Authors:** Maria V. Papadopoulou, William D. Bloomer, Howard S. Rosenzweig, Shane R. Wilkinson, Joanna Szular, Marcel Kaiser

**Affiliations:** a NorthShore University HealthSystem, Evanston, IL, United States; b Oakton Community College, Des Plaines, IL, United States; c School of Biological & Chemical Sciences, Queen Mary University of London, London, UK; d Swiss Tropical and Public Health Institute, Parasite Chemotherapy, Basel, Switzerland; e University of Basel, Basel, Switzerland

**Keywords:** Nitrotriazoles, Type I nitroreductase, Chagas disease, HAT disease, Leishmania, NTD, Neglected tropical diseases, *T. brucei*, *Trypanosoma brucei*, HAT, human African trypanosomiasis, *T. cruzi*, *Trypanosoma cruzi*, Bnz, benznidazole (*N*-benzyl-2-(2-nitro-1*H*-imidazol-1-yl)acetamide), Nfx, nifurtimox (4-(5-nitrofurfurylindenamino)-3-methylthio-morpholine-1,1-dioxide), NTR, type I nitroreductase, TcNTR, *T. cruzi* NTR, TbNTR, *T. brucei* NTR, CYP51, sterol 14α-demethylase enzyme, TcCYP51, *T. cruzi* CYP51, IC_50_, concentration for 50% growth inhibition, SI, selectivity index, SAR, structure-activity relationships, TDR, Tropical Diseases Research (http://www.who.int/tdr/en/)

## Abstract

3-Nitro-1*H*-1,2,4-triazole-based acetamides bearing a biphenyl- or a phenoxyphenyl moiety have shown remarkable antichagasic activity both *in vitro* and in an acute murine model, as well as substantial *in vitro* antileishmanial activity but lacked activity against human African trypanosomiasis. We have shown now that by inserting a methylene group in the linkage to obtain the corresponding propanamides, both antichagasic and in particular anti-human African trypanosomiasis potency was increased. Therefore, IC_50_ values at low nM concentrations against both *T. cruzi* and *T. b. rhodesiense,* along with huge selectivity indices were obtained. Although several propanamides were active against *Leishmania donovani*, they were slightly less potent than their corresponding acetamides. There was a good correlation between lipophilicity (clogP value) and trypanocidal activity, for all new compounds. Type I nitroreductase, an enzyme absent from the human host, played a role in the activation of the new compounds, which may function as prodrugs. Antichagasic activity *in vivo* was also demonstrated with representative propanamides.

## Highlights

• Novel 3-nitrotriazole-based propanamides and acetamides were synthesized.
• The propanamides demonstrated broad spectrum trypanocidal activities.
• The anti-HAT activity of propanamides was significantly better than that of acetamides.
• Lipophilicity positively affects trypanocidal activity and toxicity.
• Significant *in vivo* antichagasic activity was observed with selected propanamides.

## Graphical abstract

**Figure:**
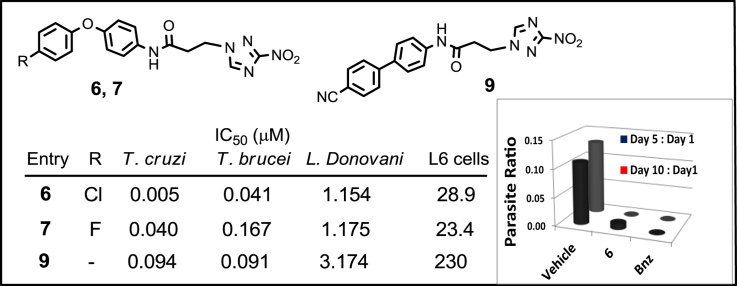

## 1 Introduction

American trypanosomiasis (Chagas disease), human African trypanosomiasis (HAT disease or sleeping sickness) and Leismaniasis are parasitic infections. They are considered neglected tropical diseases (NTD) because they constitute a major health problem in particularly poor countries around the world [1]. HAT disease (caused by *Trypanosoma brucei rhodesiense* and *T. b. gambiense*) is endemic throughout sub-Saharan Africa while Chagas disease (caused by *T. cruzi*) affects populations in South and Central America. In contrast, leishmaniasis (caused by *Leishmania* species) is prevalent in many sub-tropical and tropical regions of the world, recently expanding in non-tropical regions as HIV/AIDS co-infection [2]. These insect transmitted diseases affect more than 20 million people and are responsible for more than 110,000 deaths per year [3]. HAT disease ranks high on the list of NTD because it is fatal if untreated and the treatment options are limited. The incidence of *T. cruzi* infection has significantly declined recently, due to implementation of vector control initiatives, however, the number of cases in non-endemic sites (United States, Australia, Europe and Japan) is rising, primarily due to human and vector migration and contaminated blood transfusions [4], [5], [6].

**Fig. 1:**
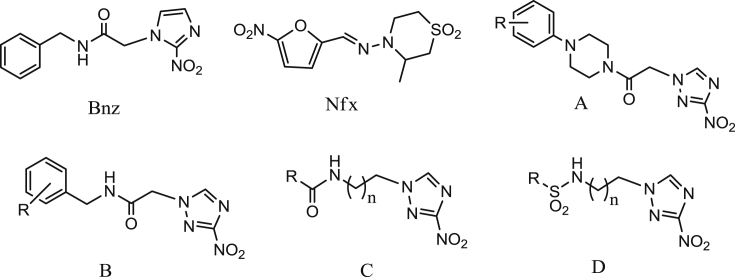
The chemical structure of Bnz, Nfx and the general structure of representative classes of 3-nitrotriazole-based trypanocidal compounds (A: piperazides, B & C: amides, and D: sulfonamides).

The treatment of neglected diseases is based on drugs with serious limitations. Thus, nifurtimox (Nfx) and benznidazole (Bnz), the two currently used medications for Chagas disease (Fig. 1) are associated with limited efficacy, severe toxicity and long treatment requirements [7], [8]. Similarly, drugs used to treat HAT and leishmaniasis are highly toxic (e.g. melarsoprol, antimonials), or require i.v. administration (e.g. melarsoprol, suramin, DFMO, antimonials) resulting in severe side effects, or are of high cost (e.g. DFMO, liposomal amphotericin B, miltefosine and paromomycin) [9], [10], [11]. Therefore, new effective, safe and affordable drugs are urgently needed for the treatment of these neglected diseases.

We have demonstrated that various chemical classes of 3-nitro-1*H*-1,2,4-triazole-based compounds, including aliphatic/aromatic amines, amides, sulfonamides, carbinols, piperazines and piperazides (some of them shown in Fig. 1) exhibit excellent antichagasic activity both *in vitro* and *in vivo,* with several analogs also showing appreciable *anti*-*T. b. rhodesiense* activity *in vitro.* [12], [13], [14], [15], [16], [17], [18] Futhermore, 3-nitrotriazole-based compounds are significantly more potent and less toxic than their 2-nitroimidazole-based counterparts [12], [13], [14], [15], [16], [17], [18], [19]. Nfx, Bnz and other nitroheterocyclics work as prodrugs, needing enzymatic activation to exert their trypanocidal activity [20], [21], [22], [23]. We have previously shown that 3-nitrotriazole-based compounds are excellent substrates of a type I nitroreductase (NTR), an oxygen-insensitive nitroreductase present in the mitochondrion of trypanosomatids and absent from most other eukaryotes [20], [21], [22], [23] and that part of the trypanocidal activity of these compounds depends on the parasite's expression of type I NTR [12], [13], [15], [16], [17], [18], [24].

Despite the failure of the antifungal drug posaconazole to treat chronic Chagas disease in clinical trials [25], there is still a great interest in developing more specific inhibitors of *T. cruzi* CYP51 (TcCYP51), the orthologous enzyme of the fungal sterol 14α-demethylase enzyme (CYP51) [26], [27], [28]. Sterol 14α-demethylase is crucial for the formation of viable membranes and the regulation of metabolic processes such as cell growth and division, not only in fungi but also in trypanosomatids [29], [30], [31], [32]. Since the triazole ring plays a significant role in CYP51 inhibition [31], we have previously evaluated 3-nitrotriazole-based amides with a linear, rigid core, as well as 3-nitrotriazole-based carbinols (fluconazole analogs) as bifunctional agents; such compounds can act as substrates for type I NTR in addition to being inhibitors of TcCYP51 [17]. These bifunctional compounds demonstrated remarkable antichagasic activity both *in vitro* and in an acute murine model [17]. A subclass of such bifunctional antitrypanosomal agents was 3-nitrotriazole-based aryloxyphenylacetamides, in which the 3-nitrotriazole ring is separated from the amidic carbonyl by one methylene-group [24]. 3-Nitrotriazole-based aryloxyphenylacetamides, besides being very potent antichagasic agents *in vitro* and *in vivo*, demonstrated also remarkable *in vitro* activity against *L. donovani* axenic amastigotes, something that was not seen before with other 3-nitrotriazole-based derivatives [24]. However, these acetamides were only moderately active against *T. b. rhodesiense*, with poor selectivity for this parasite, despite the fact that they were excellent substrates of TbNTR [24].

Therefore, in the present work we tried to further optimize the class of 3-nitrotriazole-based aryloxyphenylamides with the goal to increase their anti-HAT activity. This was obtained via linkage elongation between the nitrotriazole ring and the amidic carbonyl by inserting one additional methylene group. Thus, novel 3-nitrotriazole-based propanamides were synthesized and screened for antitrypanosomal activity. The novel propanamides were then compared side by side with the corresponding acetamides.

## 2 Results and discussion

### 2.1 Chemistry

**Scheme 1:**
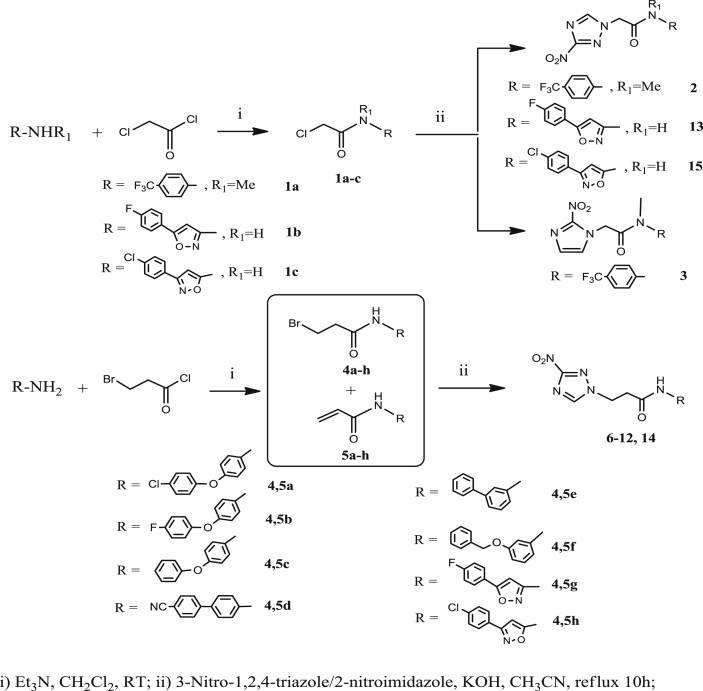
Synthesis of compounds on Table 1.

The structure of the twelve novel compounds is shown in Table 1, together with the structure of previously synthesized analogs (in orange), for comparison purposes. The synthesis of the new compounds in Table 1 is straightforward and based on well-established chemistry, outlined in Scheme 1. Thus, acetamides **2, 3, 13** and **15** were obtained by nucleophilic substitution of the appropriate chloroacetamide **1a-c** with the potassium salt of 3-nitro-1,2,4-triazole (and in the case of **3** the potassium salt of 2-nitroimidazole) under refluxing conditions. Similarly, propanamides **6–12** and **14** were obtained by nucleophilic substitution of the appropriate bromopropanamide **4a-h** with the potassium salt of 3-nitro-1,2,4-triazole under refluxing conditions. During the synthesis of bromopropanamides **4a-h,** the acrylamides **5a-h** were also formed as β-elimination byproducts, which however were not isolated due to a similar R_f_ value they share on TLC with bromopropanamides **4a-h.** Fortunately, the acrylamides **5a-h** not only did not prevent the next step but in fact they furnished as starting materials for the formation of the final propanamides through Michael addition. The final compounds were obtained in 45–78% yield.

Chloroacetamides **1a-c** and bromopropanamides **4a-h** were prepared from appropriate, commercially available arylamines and chloroacetyl chloride or 3-bromopropanoyl chloride, respectively, in the presence of triethylamine, at room temperature.

### 2.2 Biological evaluation

#### 2.2.1 Antiparasitic activity

Compounds in Table 1 were screened for anti-parasitic activity against three trypanosomatids: *T. cruzi*, *T. b. rhodesiense* and *Leishmania donovani* and compared with previously made analogs shown in orange and designated with **a**. The concentration of compound that inhibits parasite growth by 50% (IC_50_) was calculated from dose response curves for each parasite (Table 1). In addition, compounds were tested for toxicity in L6 rat skeletal myoblasts, the host cells for *T. cruzi* amastigotes, in order to calculate a selectivity index for each parasite [SI = IC_50L6_/IC_50parasite_] (Table 1). The TDR (Special Programme for Research and Training in Tropical Diseases, World Health Organization) criteria were adopted to interpret antiparasitic activity and selectivity [33].

According to the TDR criteria, all 3-nitrotriazole-based analogs were selectively ‘active’ antichagasic agents (IC_50_ of <4.0 μM, SI of ≥50), whereas the 2-nitroimidazole-based analog **3** was ‘moderately active’ against *T. cruzi* but with an unacceptable SI value (IC_50_ between 4.0 and 60 μM, SI < 50). The 3-nitrotriazole-based propanamides (**6–12**) were similar to or more potent antichagasic agents than the corresponding 3-nitrotriazole-based acetamides (**6a-11a** and **13**), most of the latter being previously evaluated (highlighted in orange and designated with **a**).

All 3-nitrotriazole-based propanamides were ‘active’ (IC_50_ < 0.5 μM) (**6**–**11**) or ‘moderately active’ (IC_50_ between 0.5 and 6.0 μM) (**12** and **14**) anti-HAT agents that displayed acceptable selectivity (SI of ≥100) towards *T. b. rhodesiense* (Table 1). Interestingly, these compounds were up to 214-fold more potent against this parasites than the corresponding acetamides (**6a-11a, 13** and **15**), with the latter structures being deemed ‘inactive’ (IC_50_ > 6.0 μM) or ‘moderately active’ at best. The increased anti-HAT activity displayed by the propanamides relative to the acetamides was always accompanied with a favorable shift in selectivity (Table 1).

The 3-nitrotriazole-based acetamide **2** was inactive against *T. b. rhodesiense*, but still more potent than the corresponding 2-nitroimidazole-based analog **3**, whereas compound **2a** of equal molecular weight with **2** was 5.8-fold more potent against *T. b. rhodesiense* than **2,** suggesting the importance of the amidic proton.

Previously we have found that 3-nitrotriazole-based aryloxy-phenylamides are selectively ‘active’ against *L. donovani* amastigotes *in vitro* (IC_50_ < 1 μM, SI ≥ 20). Increasing the distance between the nitrotriazole ring and the amidic carbonyl in compounds **6–11** and **14** sustained antileishmanial activity, although a shift to ‘moderate activity’ (IC_50_ between 1.0 and 6.0 μM) was observed for propanamides **6–8** in comparison with their acetamide-analogs **6a-8a** (Table 1).

#### 2.2.2 SAR analysis of antichagasic activity

The 3-nitrotriazole-based acetamide **2**, having an *N*-methyl substituent, was synthesized to be compared with the previously evaluated acetamide **2a**, which is unsubstituted on the amidic nitrogen but of equal molecular weight with **2**. This *N*-substitution in **2** resulted in a 1.6-fold decrease in antichagasic activity compared to **2a**, but also in a 2-fold decrease in toxicity against L6 cells, despite the fact that **2** was slightly more lipophilic than **2a** (clogP 1.99 vs 1.83). Thus, the SI of **2** was also slightly higher than that of **2a**. However, both compounds were slightly more potent antichagasic agents than Bnz (Table 1).

Acetamide **2** was also compared as antichagasic agent with its 2-nitroimidazole-based counterpart **3**. In keeping with our previous findings the former structure was shown to be 32-fold more potent towards *Trypanosoma cruzi* amastigotes than the latter. In addition, compound **3** was shown to display a higher degree of toxicity towards L6 cells compared to **2**, presumably due to its higher lipophility (Table 1). Together these initial phenotypic screens demonstrate that **3** had an unfavorable SI score (∼4) relative to that determined for **2** (∼165) with these results suggesting once again that 3-nitrotriazole-based compounds may be better antichagasic agents than 2-nitroimidazole-based analogs, at least *in vitro*.

Evaluation of the antichagasic properties of the nitrotriazole-based phenyloxyphenyl propanamides (**6–8**) with their acetamide counterparts (**6a-8a**) revealed that for each compound pairing similar potencies towards the parasite were observed (Table 1). However, due to their greater lipophilicity, propanamides displayed an increased toxicity towards L6 cells. Despite this issue, the nitrotriazole-based phenyloxyphenyl propanamides exhibited very encouraging SI values for *T. cruzi* (398–3343). The nitrotriazole-based *p*-chlorophenyloxyphenyl propanamide **6** was an extremely potent antichagasic agent with an IC_50_ of 5 nM against *T. cruzi* amastigotes, slightly more potent than the acetamide analog **6a** (IC_50_ of 8 nM). This was not the case with the fluorinated analogs **7** and **7a** and the unsubstituted pairs **8** and **8a**. Both acetamides **7a** and **8a** appeared slightly better as antichagasic agents than their propanamide-analogs **7** and **8** (Table 1). Antichagasic activity (as well as toxicity in L6 cells) decreased from the chloro-to the fluoro-, to the non-substituted phenyloxyphenyl-propanamide or acetamide, presumably as a result of decreasing lipophilicity rather than electronegativity of the substituent, since fluorine is more electronegative than chlorine (Table 1).

The growth inhibitory effect of nitrotriazole-based biphenylamides (**9, 9a**, **10** and **10a**) towards *T. cruzi* and L6 cells was also assessed (Table 1). There was about 1.5-fold increase in potency of *p*-cyanobiphenyl-propanamide **9** against *T. cruzi* parasites compared to acetamide analog **9a**. In addition, propanamide **9** was less toxic to L6 cells compared to **9a**, despite its slightly higher lipophilicity. This reduced toxicity towards L6 cells coupled with the increased potency towards *T. cruzi* parasites resulted in a better SI of **9** compared to that of **9a** (Table 1). A greater clogP value in *m*-biphenyl-propanamide **10** resulted in a 2.2-fold better antichagasic activity, with slightly increased toxicity to L6 cells compared to its acetamide analog **10a,** without, however, compromising selectivity. A drastic reduction in mammalian cell toxicity was noted for the two cyano-containing biphenylamides **9** and **9a,** being >6- and >3.5-fold less toxic towards L6 cells, respectively, relative to unsubstituted compounds **10** and **10a**. This reduction in mammalian cell toxicity, which may be related to both the electron withdrawing effect of the *p*-cyano group (inductive and resonance effects) as well as the *para-*position of the biphenyls in compounds **9** and **9a,** resulted in superior SI values compared to those obtained for compounds **10** and **10a** (Table 1).

Antichagasic activity and selectivity was improved also in the *m*-benzyloxyphenyl propanamide **11**, compared to its acetamide analog **11a**. The slightly greater lipophilicity of **11** resulted in a slightly increased toxicity towards L6 cells, without, however, compromising selectivity towards the parasite.

Finally, four novel 3-nitrotriazole based phenylisoxazole-acetamides/propanamides (**12–15**) were also evaluated for antichagasic activity *in vitro*. The *p*-fluorophenyl-isoxazole propanamide **12**, in which the *p*-fluorophenyl group is attached in the 5-position of the isoxazole ring was similarly active as an antichagasic agent with the acetamide analog **13**. Despite being more lipophilic than **13**, propanamide **12** was less toxic towards L6 cells, thus yielding a greater SI compared to **13**. In contrast, the *p*-chlorophenylisoxazole-propanamide **14**, in which the *p*-chlorophenyl group is attached in the 3-position of the isoxazole ring, was 6-fold more active as an antichagasic agent than its acetamide analog **15**. Moreover, propanamide **14** was about 10-fold more selective towards this parasite than acetamide **15**. The slightly greater clogP value of **14** may have played a role in its increased antichagasic activity but did not explain its decreased cytotoxicity towards L6 cells compared to **15**.

**Fig. 2:**
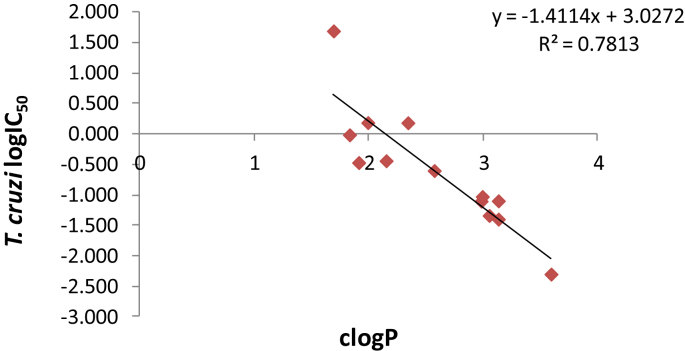
Correlation between lipophilicity (clogP values) and activity against *T. cruzi* (logIC_50_ values) of all novel compounds in Table 1.

Plotting logIC_50_ values against *T. cruzi* amastigotes *versus* clogP values for all novel compounds in Table 1, we obtained a good correlation between antichagasic activity and lipophilicity (Fig. 2). Correlation between lipophilicity and cytotoxicity towards L6 cells lead to an R^2^ of only 0.62, indicating that lipophilicity alone did not account for toxicity.

The amidic pKa value does not seem to play a role in the antichagasic activity of propanamides *versus* acetamides although more acidic amides seem to be less potent antichagasic agents (Table 1).

All novel compounds in Table 1 were 1.4- to 441-fold better antichagasic agents than Bnz (Table 1).

#### 2.2.3 SAR analysis of anti-HAT activity

Evaluation of the growth inhibitory effects of the 3-nitrotriazole-based acetamides **2** & **2a** and their 2-nitroimidazole-based analog **3** against *T. b. rhodesiense* revealed the following: firstly, all three were characterized ‘inactive’ anti-HAT agents according to the TDA criteria. Secondly, N-methylation in **2** resulted in a decreased anti-HAT activity compared to **2a** of an equal molecular weight, demonstrating the importance of the amidic hydrogen for trypanocidal activity. This decrease in the anti-HAT activity of **2** was even greater than the corresponding decrease in its antichagasic activity compared to **2a.** Finally**,** compound **3** was also significantly less potent as anti-HAT agent compared to both **2** and **2a**, showing yet again the inferior trypanocidal properties of 2-nitroimidazole-based compounds relative to their 3-nitrotriazole-based counterparts.

The phenotypic screens were then extended to evaluate the susceptibility of *T. b. rhodesiense* to 3-nitrotriazole-based propanamides (**6–12**, **14**) relative to their acetamide-analogs (**6a-11a**, **13**, **15**). For each propanamide/acetamide pairing it was clear that the former compounds were significantly more potent towards this parasite than the latter with the propanamide derivatives deemed ‘active’ (**6**–**11**) or ‘moderately active’ (**12**, **14**) anti-HAT agents relative to acetamides that were largely classed as ‘moderately active’ (**6a**-**8a**, **11a**), or ‘inactive’ (**10a**, **13**, **15**). The increased potency of the propanamides towards *T. b. rhodesiense* consistently translated to improved SI values.

Closer examination of the susceptibility data revealed that for the phenoxyphenyl-containing structures the *p*-chloro-substituted propanamide **6** was 32-fold more potent towards *T. b. rhodesiense* than its acetamide analog **6a**, the *p*-fluoro-substituted propanamide **7** was 12-fold more potent than **7a** whereas the unsubstituted propanamide **8** was 17.4-fold more potent than **8a**. A smaller shift towards lower IC_50_ values against *T. b. rhodesiense* was observed in the pair **9** and **9a**, since acetamide **9a** was already an active anti-HAT agent. Therefore the *p*-cyanobiphenyl propanamide **9** was only 3.7 fold more potent as an anti-HAT agent than the corresponding acetamide **9a** (Table 1).

An enormous shift to anti-HAT activity was observed in propanamide **10** compared to acetamide **10a**. Thus, *m*-biphenyl propanamide **10** was 214-fold more potent against *T. b. rhodesiense* compared to the corresponding acetamide **10a**, which was inactive against this parasite. This increase in the anti-HAT activity of **10** cannot be explained only by its greater lipophilicity compared to **10a**, since a similar difference in lipophilicity in the pair **6** and **6a** did not have the same effect in anti-HAT activity.

A shift from ‘moderately active’ to ‘active’ anti-HAT agent occurred in the case of the *m*-benzyloxyphenyl**-**based compounds with the propanamide **11** being 22-fold more active against *T. b. rhodesiense* than the corresponding acetamide **11a** (Table 1). A similar shift in anti-HAT activity was noted for the phenylisoxazole pairs **12** & **13** and **14** & **15**. Therefore, from the inactive *p-*fluorophenylisoxazole acetamide **13** and *p-*chlorophenylisoxazole acetamide **15** we obtained the ‘moderately active’ propanamides **12** and **14,** respectively. Propanamide **12** was 5.6-fold more active than acetamide **13** and propanamide **14** was 23-fold more active than acetamide **15** (Table 1).

**Fig. 3:**
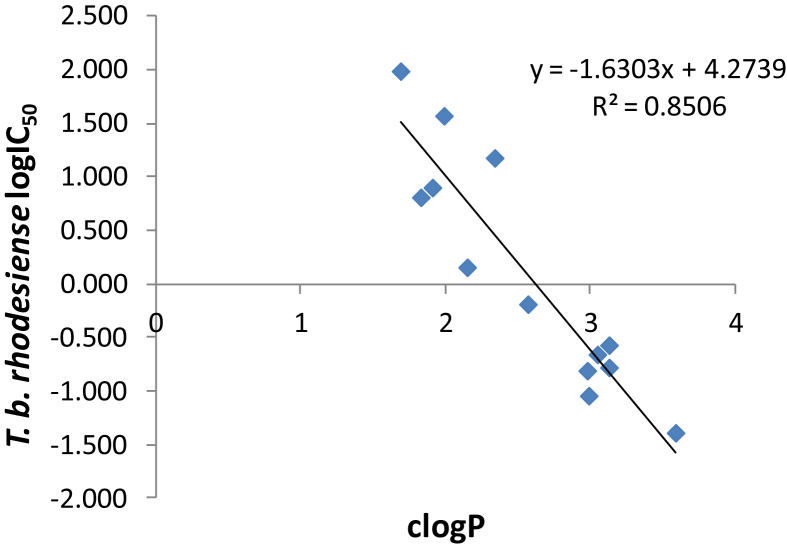
Correlation between lipophilicity (clogP values) and activity against *T. b. rhodesiense* (logIC_50_ values) of all novel compounds in Table 1.

As in the case of antichagasic activity, there was an even better correlation between lipophilicity (clogP values) and anti-HAT activity (logIC_50_ values against *T. b. rhodesiense*), with an R^2^ of 0.85 (Fig. 3). However, lipophilicity alone cannot explain the huge increase observed in the anti-HAT activity of propanamides *vs* acetamides. Interestingly, all propanamides demonstrated a larger pKa value than acetamides for the amidic ionization, which means that more acidic amides are less potent anti-HAT agents (Table 1). However, it is not clear if these *in vitro* active propanamides will exert anti-HAT activity *in vivo*, because they demonstrate relatively high PSA values (115–132), which may not permit penetration of the blood-brain barrier and thus targeting of infected tissue in the central nervous system.

#### 2.2.4 SAR analysis of antileishmanial activity

Linkage elongation between the nitrotriazole ring and the amidic carbonyl by one methylene group resulted in an ∼6- to 34-fold decreased antileishmanial activity in propanamides **6–8** compared to acetamides **6a-8a**, designating propanamides **6–8** ‘moderately active’ against this parasite. In addition, this shift to larger IC_50_ values resulted in unacceptable SI values (<20) for compounds **6** and **8**. (Table 1). A shift to decreased antileishmanial activity and an unacceptable SI value was also observed in propanamide **10** compared to acetamide-analog **10a**. Propanamide **9** was moderately active against *L. donovani* and with a good selectivity but it could not be compared to acetamide **9a** because the antileishmanial activity of the latter was not determined. In contrast, an increase in antileishmanial activity and selectivity was observed in propanamides **11, 12** and **14** compared to acetamide analogs **11a, 13** and **15**. Although the benzyloxyphenyl-propanamide **11** demonstrated moderate activity against *L. donovani* according to the criteria set, it was ca. 3-fold more potent than the acetamide analog **11a**, with both exhibiting acceptable SI values (Table 1).

In the case of the *p*-fluorophenylisoxazoles **12** and **13**, we can observe that the IC_50_ value of propanamide **12** was slightly lower than that of acetamide **13**, although both were deemed ‘inactive’ against *L. donovani*. In contrast, the *p*-chlorophenylisoxazole-propanamide **14** was ‘moderately active’ against *L. donovani* and with an acceptable selectivity, whereas the corresponding acetamide **15** was ‘inactive’ against this parasite (Table 1).

Once again, there was a good correlation between lipophilicity and antileishmanial activity (R^2^ = 0.78) for the novel compounds (data not shown) but in this case increased antileishmanial activity was associated with increased amidic acidity in pairs **6-6a, 7-7a, 8-8a**, and **10**–**10a** while decreased antileishmanial activity was associated with increased acidity in pairs **11**–**11a, 12–13** and **14–15** (Table 1).

#### 2.2.5 Involvement of type I nitroreductase

**Fig. 4:**
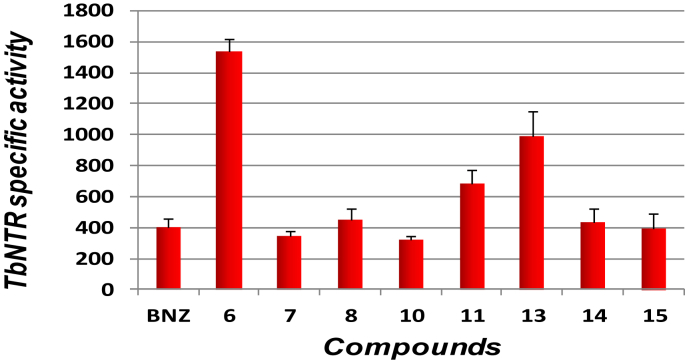
Activity of TbNTR toward different 3-nitrotriazole-based compounds. The TbNTR specific activity expressed as nmol NADH oxidized min^−1^mg^−1^ of purified his-tagged protein was assessed using representative 3-nitrotriazoles as substrates and the values shown are the means from three experiments ± standard deviation. The activity obtained when using benznidazole (BNZ) as substrate is also shown.

To elucidate the mechanism of action of the novel compounds in Table 1, representative 3-nitrotriazole-based propanamides (**6–8**, **10, 11, 14**) and acetamides (**13, 15**) were evaluated as substrates of purified, recombinant TbNTR and compared to benznidazole (Fig. 4). Enzyme specific activity was measured as oxidized NADH (nmol) per min per mg of protein. Compounds **9** and **12** were also analyzed but these precipitated in the assay buffer and no activity could be determined. All remaining compounds were shown to be substrates of TbNTR, with compounds **6**, **11** and **13** yielding specific enzyme activity values up to ∼4-fold greater than benznidazole. In the only propanamide/acetamide pairing tested (**14**, **15**), TbNTR metabolized both compounds at equivalent rates despite the former exhibiting greater anti-HAT activity [24]. As we have reported before with other classes of 3-nitrotriazole-based antitrypanosomal compounds, no correlation between anti-HAT activity and TbNTR specific activity was observed [12], [13], [15], [16], [17], [18].

When the above biochemical tests were extended to phenotypic screens, BSF *T. b. brucei* parasites engineered to express elevated levels of TbNTR were shown to be more susceptible to the compounds under study than controls, thus indicating that within the parasite the nitrotriazoles are functioning as TbNTR activated prodrugs (Table 2). Intriguingly, the ratio of the IC_50_ values between the recombinant line and wild type did vary from agent to agent. For some compounds (e.g. **6** and **7**) the fold difference between the two lines was low (∼2-fold) with these structures generally exhibiting a moderate *anti*-*T. b. brucei* activity against wild type parasites (IC_50_ values of ∼0.65 μM). In contrast, the TbNTR overexpressing line was shown to be 10- to 25-fold more susceptible to other agents (e.g. **10, 11**, and **15**) that exhibit a lower potency against control lines to start with (IC_50_ values of 4–8 μM). Together this indicates that for compounds which are less effective against wild type *T. b. brucei* it is the activation step that appears to limit their trypanocidal potential. When the rate limiting reaction is alleviated, in this case through the over expression of the trypanosomal type I NTR activity, then most of the nitrotriazoles tested exhibit equivalent potencies towards the parasite at which point other downstream factors may now be affecting the *anti*-*T. b. brucei* activity.

#### 2.2.6 In vivo evaluation

**Fig. 5:**
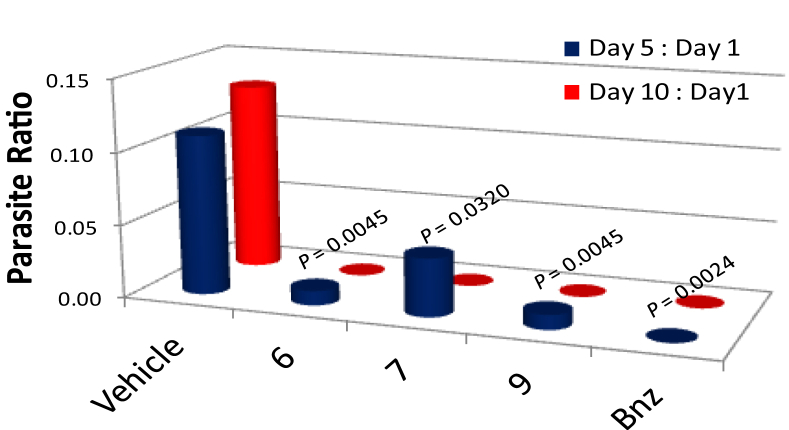
*In vivo* evaluation of the antichagasic efficacy of compounds **6, 7, 9** and Bnz in the acute murine model. Compounds **6** and **7** were administered (i.p.) at 13 mg/kg/day while compound **9** and Bnz were administered at 15 mg/kg/day (i.p.) up to 10 consecutive days. Parasite ratios were calculated on day 5 and 10. The *P* values between each treated group and control group for 5-day treatment are shown on the graph. The *P* value was 0.0001 between each treated group and control group for 10-day treatment. Groups of 5 mice/group were used.

Compounds **6, 7** and **9** were chosen for *in vivo* evaluation, based on their high *in vitro* potency and selectivity against *Trypanosoma cruzi* amastigotes (Table 1). A fast luminescence assay was used in an acute infected murine model, in which transgenic *T. cruzi* parasites expressing firefly luciferase were injected in 5 week-old Balb/c mice. Mice were injected with D-luciferin before imaging. Groups of 5 mice/group were treated i.p. for 10 consecutive days with each compound, at 13 or 15 mg/kg/day. In our previous work with 3-nitrotriazole-based rigid amides we have seen very good *in vivo* antichagasic activity at daily doses of 15 mg/kg [17], therefore we decided to use a slightly lower dose for compounds **6** and **7** which demonstrated IC_50_ values against *T. cruzi* amastigotes at low nM concentrations (Table 1). Bnz was used in parallel as a positive control at 15 mg/kg/day (i.p.). The mean ratio of parasite levels was calculated after 5 and 10 days of treatment. The data are summarized in Fig. 5. All tested compounds, including Bnz, reduced the parasite load to undetectable levels after 10 days of treatment with statistical significance (*p* < 0.0001). Statistically significant reduction in parasitic level (compared to untreated control) was also observed after 5-day treatment with each compound. However, mice in the group treated with compound **6** did not look very healthy, although no deaths occurred. Compound **6** demonstrates the lowest IC_50_ value in L6 cells among all analogs tested (Table 1), and perhaps a smaller dose should have been used in mice. No toxicity symptoms were observed with compounds **7** and **9**.

## 3 Conclusions

3-Nitrotriazole-based propanamides with a broad spectrum of antitrypanosomal activity were synthesized. The *in vitro* antichagasic activity of propanamides was excellent and comparable to that of the corresponding acetamides. However, their *in vitro* anti-HAT activity was 4- to 214-fold greater than that of the corresponding acetamide analogs. With regard to the antileishmanial activity, a decrease was observed for some propanamides whereas a comparable or greater activity was observed for others in comparison with their acetamide-analogs. Antitrypanosomal activity was in good correlation with lipophilicity. Decreased amidic acidity tended to favor anti-HAT activity whereas increased amidic acidity tended to favor antileishmanial activity in most of the compounds. 3-Nitrotriazole-based propanamides are good substrates for type I NTR, but no better than the corresponding acetamides. There was no correlation between enzymatic specific activity and trypanocidal activity, presumably due to the existence of additional targets in the trypanosome, permeability issues with regard to mitochondrion, compound stability and pharmacokinetic factors in general. Such lack of correlation was observed and discussed before with other classes of 3-nitrotriazole-based antitrypanosomal agents [12], [13], [15], [16], [17], [18]. With regard to potential additional targets, it was demonstrated that in addition to type I NTR activation, Bnz toxicity against *Trypanosoma cruzi* is mediated by various other mechanisms including thiol depletion (by Bnz reduction products) [34], interferon-γ mediated activation of the immune system [35], and NADH-fumarate inhibition [36]. Therefore, further investigation is needed to elucidate if additional enzymes are involved in the mechanism of action of 3-nitrotriazole-based propanamides as well as the role of CYP51.

Representative 3-nitrotriazole-based propanamides demonstrated very good antichagasic activity in an acute *T. cruzi* murine model, but further investigation is required to see if such compounds can provide cures in the chronic model, taking into account also toxicity issues.

## 4 Experimental

### 4.1 Chemistry

#### 4.1.1 General

All starting materials and solvents were purchased from Sigma-Aldrich (Milwaukee, WI), were of research-grade quality and used without further purification. Solvents used were anhydrous and the reactions were carried out under a nitrogen atmosphere and exclusion of moisture. Melting points were determined by using a Mel-Temp II Laboratory Devices apparatus (Holliston, MA) and are uncorrected. Proton NMR spectra were obtained on a Varian Inova-500 or an Agilent Hg-400 spectrometer at 500 or 400 MHz, respectively, and are referenced to Me_4_Si or to the corresponding solvent, if the solvent was not CDCl_3_. High-resolution electrospray ionization (HRESIMS) mass spectra were obtained on an Agilent 6210 LC-TOF mass spectrometer at 11,000 resolution. Thin-layer chromatography was carried out on aluminum oxide N/UV_254_ or polygram silica gel G/UV_254_ coated plates (0.2 mm, Analtech, Newark, DE). Chromatography was carried out on preparative TLC alumina GF (1000 μm) or silica gel GF (1500 μm) plates (Analtech). All final compounds were purified by preparative TLC chromatography on silica gel plates and also checked by HPLC (Agilent 1100) coupled to mass spectrometry (MS) and ultraviolet (UV) absorbance detectors. Purity was determined by comparing the area under the curve of the test compound peak to the combined areas of all peaks in the chromatogram, excluding DMSO and any peaks appearing in blank runs. Priority reporting was given to the UV traces (since mass spectrometer response can vary widely depending on a given compound's ease of ionization). Purity of compounds lacking UV activity was reported using mass spectrometry detection. All final compounds were 100% pure, according to HPLC-UV analysis.

#### 4.1.2 Synthesis of precursors **1a-c** and **(4** + **5)a-h**

Compounds **1a-c** and **(4** + **5)a-h** were synthesized as follows: In a dichloromethane solution (3 mL) of chloroacetylchloride (1.1 eq) or 3-bromopropanoyl chloride (1.1 eq), a dichloromethane solution (8–10 mL) of the appropriate amine (1 eq, usually 200–300 mg) and triethylamine (1.1 eq) was added dropwise and the reaction mixture was stirred overnight at room temperature under a nitrogen atmosphere. The reaction mixture was evaporated and redissolved in ethyl acetate. The inorganic salts were filtered away and the filtrate was evaporated to give the desire product in 80–85% yield, which however, in most cases contained the corresponding acrylamide (**5a-h**) as a β-elimination byproduct. In certain cases, chromatography was applied to separate the bromides from the acrylamides for identification purposes, however in most cases the unpurified mixture was used for the next step, since both precursors lead to the desired final product. The spectroscopic data of compounds **1a-c** and **4a-h** (as well as of some acrylamide byproducts) are provided in the Supplementary Material.

#### 4.1.3 General synthesis of nitro(triazolyl/imidazolyl)acetamides and propanamides **2, 3, 6**–**15**

The potassium salt of 3-nitro-1,2,4-triazole or 2-nitroimidazole (1 eq, usually 100 mg) was formed in CH_3_CN (6–10 mL), by refluxing with KOH (1.2 eq) for 30 min. To this suspension **1a-c** or **(4** + **5)a-h** (1.1 eq) was added and the reaction mixture was refluxed under a nitrogen atmosphere for 10 h. The reaction mixture was checked by TLC for completion of the reaction and the solvent was evaporated. The residue was dissolved in ethyl acetate and the inorganic salts were filtered away. Upon preparative TLC (silica gel; ethyl acetate-petroleum ether), the desired product was obtained usually as a powder. Purity was checked also by HPLC and it was 100%.

##### 4.1.3.1 *N*-methyl-2-(3-nitro-1H-1,2,4-triazol-1-yl)-*N*-(4-(trifluoromethyl)phenyl)acetamide **(2)**

White microcrystals (216 mg, 64%): mp 133–134 °C; ^1^H NMR (400 MHz, CDCl_3_) δ: 8.35 (s, 1H, triazolic), 7.82 (d, *J* = 8.0 Hz, 2H, phenylic), 7.47 (d, *J* = 8.4 Hz, 2H, phenylic), 4.83 (s, 2H, COCH_2_-), 3.37 (s, 3H, CH_3_). HRESIMS calcd for C_12_H_11_F_3_N_5_O_3_ and C_12_H_10_F_3_N_5_NaO_3_ *m/z* [M+H]^+^ and [M+Na]^+^ 330.0809 and 352.0628, found 330.0806 and 352.0619.

##### 4.1.3.2 *N*-methyl-2-(2-nitro-1H-imidazol-1-yl)-*N*-(4-(trifluoromethyl)phenyl)acetamide **(3)**

Thick oil that solidified to yellowish crystals (180 mg, 62%): mp 45–47 °C; ^1^H NMR (400 MHz, CDCl_3_) δ: 7.82 (d, *J* = 8.0 Hz, 2H, phenylic), 7.55 (d, *J* = 8.4 Hz, 2H, phenylic), 7.18 (s, 1H, imidazolic), 7.02 (s, 1H, imidazolic), 4.86 (s, 2H, COCH_2_-), 3.36 (s, 3H, CH_3_). HRESIMS calcd for C_13_H_12_F_3_N_4_O_3_ and C_13_H_11_F_3_N_4_NaO_3_ *m/z* [M+H]^+^ and [M+Na]^+^ 329.0856 and 351.0675, found 329.0849 and 351.0669.

##### 4.1.3.3 *N*-(4-(4-chlorophenoxy)phenyl)-3-(3-nitro-1H-1,2,4-triazol-1-yl)propanamide **(6)**

Off white powder (126 mg, 48%): mp 138–140 °C; ^1^H NMR (400 MHz, CD_3_COCD_3_) δ: 9.37 (br s, 1H, amidic), 8.66 (s, 1H, triazolic), 7.64 (d, *J* = 9.2 Hz, 2H, phenylic), 7.37 (d, *J* = 9.2 Hz, 2H, phenylic), 6.98 (dd, *J* = 9.2, 6.4 Hz, 4H, phenylic), 4.74 (t, *J* = 6.4 Hz, 2H, COCH_2_CH_2_-), 3.13 (t, *J* = 6.4 Hz, 2H, COCH_2_CH_2_-). HRESIMS calcd for C_17_H_15_ClN_5_O_4_ and C_17_H_14_ClN_5_NaO_4_ *m/z* [M+H]^+^ and [M+Na]^+^ 388.0807, 390.0784 and 410.0627, 412.0604, found 388.0806, 390.0786 and 410.0628, 412.0605.

##### 4.1.3.4 *N*-(4-(4-fluorophenoxy)phenyl)-3-(3-nitro-1H-1,2,4-triazol-1-yl)propanamide (**7**)

Off white microcrystals (174 mg, 58%): mp 124–125 °C; ^1^H NMR (400 MHz, CD_3_COCD_3_) δ: 9.34 (br s, 1H, amidic), 8.66 (s, 1H, triazolic), 7.61 (d, *J* = 9.2 Hz, 2H, phenylic), 7.13 (t, *J* = 9.2 Hz, 2H, phenylic), 7.024–6.99 (m, 2H, phenylic), 6.94 (d, *J* = 9.2 Hz, 2H, phenylic), 4.74 (t, *J* = 6.4 Hz, 2H, COCH_2_CH_2_-), 3.12 (t, *J* = 6.4 Hz, 2H, COCH_2_CH_2_-). HRESIMS calcd for C_17_H_15_FN_5_O_4_ and C_17_H_14_FN_5_NaO_4_ *m/z* [M+H]^+^ and [M+Na]^+^ 372.1103 and 394.0922, found 372.1104 and 394.0924.

##### 4.1.3.5 3-(3-Nitro-1H-1,2,4-triazol-1-yl)-*N*-(4-phenoxyphenyl)propanamide **(8)**

Light yellow microcrystals (172 mg, 57%): mp 108–110 °C; ^1^H NMR (400 MHz, CD_3_COCD_3_) δ: 9.35 (br s, 1H, amidic), 8.66 (s, 1H, triazolic), 7.62 (d, *J* = 8.8 Hz, 2H, phenolic), 7.35 (dt, *J* = 7.6, 1.2 Hz, 2H, phenolic), 7.09 (t, *J* = 7.6 Hz, 1H, phenolic), 6.96 (dd, *J* = 9.6, 2.0 Hz, 4H, phenolic), 4.74 (t, *J* = 6.4 Hz, 2H, COCH_2_CH_2_-), 3.13 (t, *J* = 6.4 Hz, 2H, COCH_2_CH_2_-). HRESIMS calcd for C_17_H_16_N_5_O_4_ and C_17_H_15_N_5_NaO_4_ *m/z* [M+H]^+^ and [M+Na]^+^ 354.1197 and 376.1016, found 354.120 and 376.1018.

##### 4.1.3.6 *N*-(4′-cyano-[1,1′-biphenyl]-4-yl)-3-(3-nitro-1H-1,2,4-triazol-1-yl)propanamide **(9)**

Off white microcrystals (120 mg, 48%): mp 200–204 °C (dec.); ^1^H NMR (400 MHz, CD_3_COCD_3_) δ: 9.50 (br s, 1H, amidic), 8.69 (s, 1H, triazolic), 7.87–7.69 (m, 8H, phenylic), 4.76 (t, *J* = 6.4 Hz, 2H, COCH_2_CH_2_-), 3.18 (t, *J* = 6.4 Hz, 2H, COCH_2_CH_2_-). HRESIMS calcd for C_18_H_15_N_6_O_3_ and C_18_H_14_N_6_NaO_3_ *m/z* [M+H]^+^ and [M+Na]^+^ 363.120 and 385.102, found 363.1205 and 385.1017.

##### 4.1.3.7 *N*-([1,1′-biphenyl]-3-yl)-3-(3-nitro-1H-1,2,4-triazol-1-yl)propanamide **(10)**

White powder (121 mg, 65%): mp 174–176 °C; ^1^H NMR (400 MHz, CD_3_COCD_3_) δ: 9.42 (br s, 1H, amidic, 8.68 (s, 1H, triazolic), 7.96 (d, *J* = 2.0 Hz, 1H, phenylic), 7.62–7.34 (m, 8H, phenylic), 4.76 (t, *J* = 6.4 Hz, 2H, COCH_2_CH_2_-), 3.17 (t, *J* = 6.4 Hz, 2H, COCH_2_CH_2_-). HRESIMS calcd for C_17_H_16_N_5_O_3_ and C_17_H_15_N_5_NaO_3_ *m/z* [M+H]^+^ and [M+Na]^+^ 338.1248 and 360.1067, found 338.1251 and 360.1071.

##### 4.1.3.8 *N*-(3-(benzyloxy)phenyl)-3-(3-nitro-1H-1,2,4-triazol-1-yl)propanamide **(11)**

White microcrystals (81 mg, 45%): mp 119–120 °C; ^1^H NMR (400 MHz, CD_3_COCD_3_) δ: 9.30 (br s, 1H, amidic), 8.66 (s, 1H, triazolic), 7.48–7.33 (m, 6H, phenylic), 7.19 (t, *J* = 8.4 Hz, 1H, phenylic), 7.09 (d, *J* = 8.8 Hz, 1H, phenylic), 6.72 (dd, *J* = 8.0, 1.6 Hz, 1H, phenylic), 5.08 (s, 2H, benzylic), 4.73 (t, *J* = 6.8 Hz, 2H, COCH_2_CH_2_-), 3.12 (t, *J* = 6.4 Hz, 2H, COCH_2_CH_2_-). HRESIMS calcd for C_18_H_18_N_5_O_4_ and C_18_H_17_N_5_NaO_4_ *m/z* [M+H]^+^ and [M+Na]^+^ 368.1353 and 390.1173, found 368.1356 and 390.1178.

##### 4.1.3.9 *N*-(5-(4-fluorophenyl)isoxazol-3-yl)-3-(3-nitro-1H-1,2,4-triazol-1-yl)propanamide **(12)**

White powder (197 mg, 75%): mp 215 °C (dec); ^1^H NMR (400 MHz, CD_3_COCD_3_) δ: 10.23 (br s, 1H, amidic), 8.69 (s, 1H, triazolic), 7.95 (dd, *J* = 9.2, 5.6 Hz, 2H, phenylic), 7.32 (t, *J* = 9.2 Hz, 2H, phenylic), 7.27 (s, 1H, isoxazolic), 4.79 (t, *J* = 6.4 Hz, 2H, COCH_2_CH_2_-), 3.29 (t, *J* = 6.4 Hz, 2H, COCH_2_CH_2_-). HRESIMS calcd for C_14_H_12_FN_6_O_4_ and C_14_H_11_FN_6_NaO_4_ *m/z* [M+H]^+^ and [M+Na]^+^ 347.0899 and 369.0718 found 347.0906 and 369.0724.

##### 4.1.3.10 *N*-(5-(4-fluorophenyl)isoxazol-3-yl)-2-(3-nitro-1H-1,2,4-triazol-1-yl)acetamide **(13)**

White powder (120 mg, 46%): mp > 220 °C; ^1^H NMR (400 MHz, CD_3_COCD_3_) δ: 10.67 (br s, 1H, amidic), 8.78 (s, 1H, triazolic), 7.97 (dd, *J* = 9.2, 5.6 Hz, 2H, phenylic), 7.33 (t, *J* = 8.8 Hz, 2H, phenylic), 7.28 (s, 1H, isoxazolic), 5.59 (s, 2H, COCH_2_-). HRESIMS calcd for C_13_H_10_FN_6_O_4_ and C_13_H_9_FN_6_NaO_4_ *m/z* [M+H]^+^ and [M+Na]^+^ 333.0742 and 355.0562, found 333.0743 and 355.0564.

##### 4.1.3.11 *N*-(3-(4-chlorophenyl)isoxazol-5-yl)-3-(3-nitro-1H-1,2,4-triazol-1-yl)propanamide **(14)**

Off white microcrystals (220 mg, 78%): mp 207–209 °C; ^1^H NMR (400 MHz, CD_3_COCD_3_) δ: 10.85 (br s, 1H, amidic), 8.69 (s, 1H, triazolic), 7.90 (d, *J* = 8.4 Hz, 2H, phenylic), 7.54 (d, *J* = 8.4 Hz, 2H, phenylic), 6.72 (s, 1H, isoxazolic), 4.81 (t, *J* = 6.4 Hz, 2H, COCH_2_CH_2_-), 3.32 (t, *J* = 6.4 Hz, 2H, COCH_2_CH_2_-). HRESIMS calcd for C_14_H_12_ClN_6_O_4_ and C_14_H_11_ClN_6_NaO_4_ *m/z* [M+H]^+^ and [M+Na]^+^ 363.0603, 365.0579 and 385.0423, 387.0398 found 363.0606, 365.0581 and 385.0422, 387.0392.

##### 4.1.3.12 *N*-(3-(4-chlorophenyl)isoxazol-5-yl)-2-(3-nitro-1H-1,2,4-triazol-1-yl)acetamide **(15)**

Pinkish powder (130 mg, 71%): mp 215–217 °C; ^1^H NMR (400 MHz, CD_3_COCD_3_) δ: 11.26 (br s, 0.5H, amidic), 8.78 (s, 1H, triazolic), 7.91 (d, *J* = 8.8 Hz, 2H, phenylic), 7.54 (d, *J* = 8.8 Hz, 2H, phenylic), 6.78 (s, 1H, isoxazolic), 5.62 (s, 2H, COCH_2_-). HRESIMS calcd for C_13_H_10_ClN_6_O_4_ and C_13_H_9_ClN_6_NaO_4_ *m/z* [M+H]^+^ and [M+Na]^+^ 349.0447, 351.0422 and 371.0266, 373.0241 found 349.0448, 351.0422 and 371.0264, 373.0239.

### 4.2 Biological evaluation

#### 4.2.1 In vitro screening

*In vitro* activity against *T. cruzi*, *T. b. rhodesiense, L. donovani* and cytotoxicity assessment using L6 cells (rat skeletal myoblasts) was determined using a 96-well plate format as previously described [37]. Data were analyzed with the graphic program Softmax Pro (Molecular Devices, Sunnyvale, CA, USA), which calculated IC_50_ values by linear regression from the sigmoidal dose inhibition curves.

#### 4.2.2 In vitro T. brucei antiproliferating assays and susceptibility studies

The BSF of *T. brucei* parasites were seeded at 1 × 10^3^ mL^−1^ in 200 μL of growth medium containing different concentrations of nitroheterocycle. Where appropriate, induction of the TbNTR was carried out by adding tetracycline (1 μg/mL). After incubation for 3 days at 37 °C, 20 μL of Alamar blue was added to each well and the plates incubated for a further 16 h. The cell density of each culture was determined as described before [20] and the IC_50_ established.

#### 4.2.3 Enzymatic activity studies with type I TbNTR

Recombinant TbNTR was prepared and assayed as previously described [38], [39]. The specific activity of purified his-tagged TbNTR was assessed spectrophotometrically at 340 nm using various nitrotriazole substrates (100 μM) and NADH (100 μM) and expressed as nmol NADH oxidized min^−1^ mg^−1^ of enzyme.

#### 4.2.4 In vivo antichagasic activity assessment of selected compounds

For *in vivo* studies, a Brazilian strain trypomastigotes from transgenic *T. cruzi* parasites expressing firefly luciferase were used as described before [15]. Briefly, parasites (10^5^ trypomastigotes) were injected in 5 week-old Balb/c mice (Taconic) and three days later mice were anesthesized by inhalation of isofluorane, followed by an injection with 150 mg/kg of D-luciferin potassium-salt in PBS. Mice were imaged 5–10 min after injection of luciferin with an IVIS 100 (Xenogen, Alameda, CA) and the data acquisition and analysis were performed with the software LivingImage (Xenogen) as described before [40]. Treatment with test compounds started 4 days post infection at 13 or 15 mg/kg/day × 10 days, given i.p. The vehicle control was 2% methylcellulose +0.5% Tween 80 and groups of 5 mice/group were used. Mice were imaged after 5 and 10 days of treatment. The ratio of parasite levels was calculated for each animal dividing the luciferase signal after treatment by the luciferase signal on the first imaging (before treatment). Mean values of all animals in each group ± SD were used for plotting.

## Figures and Tables

**Table 1:**
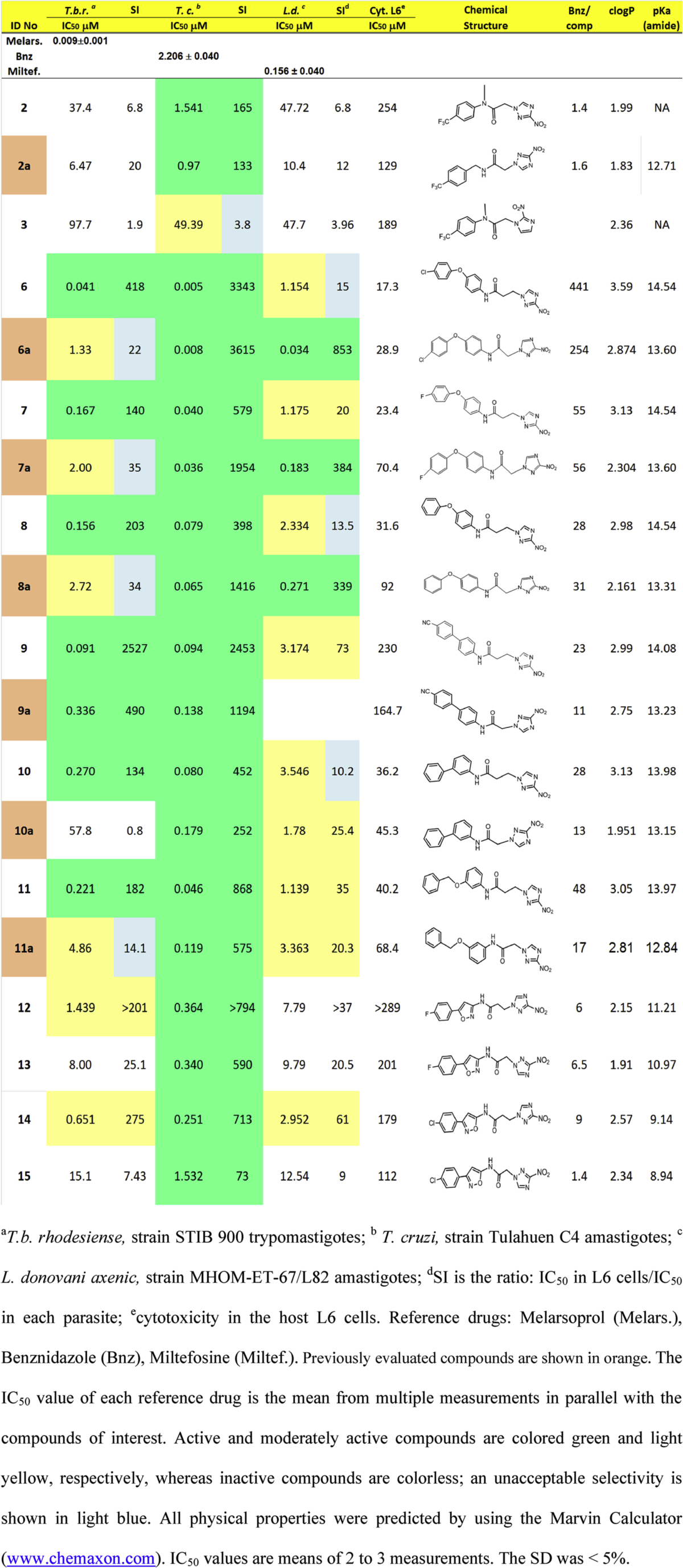
*In vitro* antiparasitic activity, host toxicity and physical properties of tested compounds.

**Table 2:** Susceptibility of bloodstream form *T.b. brucei* with altered levels of NTR towards selected nitrotriazoles.

Compound	IC_50_ value (μM) *T.b. brucei*			Ratio –tet/+tet
	Wild type	TbNTR (-tet)	TbNTR (+tet)	
**nfx**	3.98 ± 0.15	6.359 ± 0.119	0.869 ± 0.046	7.3
**6**	0.64 ± 0.02	0.62 ± 0.03	0.34 ± 0.00	1.8
**7**	0.57 ± 0.05	0.68 ± 0.07	0.33 ± 0.03	2.1
**8**	0.14 ± 0.01	1.47 ± 0.03	0.41 ± 0.01	3.6
**9**	0.12 ± 0.01	0.55 ± 0.04	0.12 ± 0.01	4.7
**10**	6.66 ± 0.92	5.58 ± 0.62	0.23 ± 0.02	24.6
**11**	4.25 ± 0.16	3.70 ± 0.58	0.36 ± 0.02	10.4
**12**	0.76 ± 0.10	1.61 ± 0.10	0.40 ± 0.00	4.1
**13**	26.61 ± 6.02	–	–	
**14**	0.27 ± 0.05	0.91 ± 0.03	0.29 ± 0.02	4.2
**15**	8.65 ± 0.49	13.27 ± 1.40	1.23 ± 0.06	10.8

Growth-inhibitory effect as judged by IC_50_ values (in μM) of selected 3-nitroriazoles on *T.b. brucei* wild type and recombinant parasites expressing wild type [TbNTR (-tet)] or elevated [TbNTR (+tet)] levels of TbNTR. Data are means from 4 experiments ± standard deviation. Nifurtimox (nfx) was used as control. Only compounds exhibiting an IC_50_ < 10 μM in wild type parasites were screened against the recombinant line.

## Appendix A Supplementary data

### Mol Files

The following ZIP file contains the MOL files of the most important compounds referred to in this article.

MOL file. ZIP file containing the MOL files of the most important compounds in this article.
