# Supplementary material for: Nitrotriazole-based acetamides and propanamides with broad spectrum antitrypanosomal activity
Source: Eur J Med Chem. 2016 Nov 10;123:895–904. doi: 10.1016/j.ejmech.2016.08.002 (PMC5049494; doi:10.1016/j.ejmech.2016.08.002)
Supplement: Supplementary file 1 [file mmc1.docx]

**Nitro****triazole-based Acetamides and Propanamides** **with Broad Spectrum Antitrypanosomal Activity**

Maria V. Papadopoulou,*^[a]^ William D. Bloomer,^[a]^ Howard S. Rosenzweig,^[b]^ Shane R. Wilkinson,^[c]^ Bhawana Gurung, ^[c]^ Joanna Szular^[c]^ and Marcel Kaiser^[d, e]^

^a^*NorthShore University HealthSystem, Evanston, IL, US;* ^b^*Oakton Community College, Des Plaines, IL, US;* ^c^ *School of Biological & Chemical Sciences, Queen Mary University of London, London, UK;* ^d^*Swiss Tropical and Public Health Institute, Parasite Chemotherapy, Basel, Switzerland;* ^e^*University of Basel, Basel, Switzerland.*

**Spectroscopic data and references of precursors 1a-c and 4a-h.**

*2-chloro-N-methyl-N-(4-(trifluoromethyl)phenyl)acetamide (NS-252)* ***(1a)****^1^***:** Colorless oil (86 %): ^1^H NMR (400 MHz, CDCl_3_) δ: 7.73 (d, *J*=8.8 Hz, 2H), 7.41 (d, *J*=8.8 Hz, 2H), 3.86 (s, 2H), 3.35 (s, 3H). HRESIMS calcd for C_10_H_10_F_3_ClNO *m/z* [M+H]^+^ 252.0403, 254.0370 found 252.0396, 254.0372.

*2-chloro-N-(5-(4-fluorophenyl)isoxazol-3-yl)acetamide (NS-270)* ***(1b)***: Off white powder (60%). ^1^H NMR (400 MHz, CDCl_3_) δ: 8.91 (br s, 1H), 7.80 (dd, *J*=8.5, 5.2 Hz, 2H), 7.20-7.15 (m, 3H), 4.24 (s, 2H). HRESIMS calcd for C_11_H_8_ClFN_2_NaO_2_ *m/z* [M+Na]^+^ 277.0151, 279.0124 found 277.0151, 279.0116.

*2-chloro-N-(3-(4-chlorophenyl)isoxazol-5-yl)acetamide (NS-269)* ***(1c)****^2^*: Off white powder (45%). ^1^H NMR (400 MHz, CDCl_3_) δ: 9.06 (br s, 1H), 7.76 (d, *J*=8.8 Hz, 2H), 7.44 (d, *J*=8.8 Hz, 2H), 6.76 (s, 1H), 4.28 (s, 2H). HRESIMS calcd for C_11_H_8_Cl_2_N_2_NaO_2_ *m/z* [M+Na]^+^ 292.9861, 294.9830 found 292.9858, 294.9829.

*3-bromo-N-(4-(4-chlorophenoxy)phenyl)propanamide (NS-257)* ***(4a)****^3^* *and N-(4-(4-chlorophenoxy)phenyl) acrylamide* ***(5a)****^4^****.*** Both compounds were found together at approximately 1:4 ratio as a pinkish powder (87%): ^1^H NMR (400 MHz, CDCl_3_) δ: (**4a**): 7.56 (d, *J*=8.4 Hz, 2H), 7.28 (d, *J*=8.8 Hz, 2H), 7.22 (br s, 1 H), 6.98 (d, *J*=8.8 Hz, 2H), 6.92 (d, *J*=8.8 Hz, 2H), 3.90 (t, *J*=6.4 Hz, 2H), 2.82 (t, *J*=6.4 Hz, 2H). (**5a**): 7.56 (d, *J*=8.4 Hz, 2H), 7.28 (d, *J*=8.8 Hz, 2H), 6.98 (d, *J*=8.8 Hz, 2H), 6.92 (d, *J*=8.8 Hz, 2H), 6.45 (d, *J*=16.8 Hz, 1H), 6.24 (dd, *J*=16.8, 10.0 Hz, 1H), 5.79 (d, *J*=11.6 Hz, 1H). HRESIMS calcd for C_15_H_14_BrClNO_2_ (**4a**)  *m/z* [M+H]^+^ 353.9896, 355.9880 found 353.9892, 355.9881. HRESIMS calcd for C_15_H_12_ClNNaO_2_ (**5a**)  *m/z* [M+Na]^+^ 296.0451, 298.0425 found 296.0449, 298.0424.

*3-bromo-N-(4-(4-fluorophenoxy)phenyl)propanamide (NS-258)* ***(4b)****^3^* *and* *N-(4-(4-fluorophenoxy)phenyl) acrylamide* ***(5b)*:** Pinkish crystals as mixture with **5b** at approximately 1:4 ratio (81 %): ^1^H NMR (400 MHz, CDCl_3_) δ: (**4b**): 7.55-6.94 (m, 9H), 3.62 (t, *J*=4.4 Hz, 2H), 3.13 (t, *J*=4.4 Hz, 2H). (**5b**): 7.55-6.94 (m, 8H), 6.44 (dd, *J*=16.8, 1.2 Hz, 1H), 6.24 (dd, *J*=16.8, 10.0 Hz, 1H), 5.78 (dd, *J*=10.0, 1.2 Hz, 1H). HRESIMS calcd for C_15_H_13_BrFNNaO_2_ (**4b**) *m/z* [M+Na]^+^ 360.0011, 361.9990 found 360.0009, 361.9992. HRESIMS calcd for C_15_H_12_FNNaO_2_ (**4b**) *m/z* [M+Na]^+^ 280.0749 found 280.0750.

*3-bromo-N-(4-phenoxyphenyl)propanamide (NS-259)* ***(4c)****^5^*: Off white powder (78 %), containing *N*-(4-phenoxyphenyl)acrylamide **5c**^4^ (2:1): ^1^H NMR (400 MHz, CDCl_3_) δ: (**4c**): 7.48 (d, *J*=8.8 Hz, 2H), 7.33 (t, *J*=7.8 Hz, 2H), 7.20 (br s, 1H), 7.09 (t, *J*=7.2 Hz, 1H), 7.01-6.98 (m, 4H), 3.73 (t, *J*=6.4 Hz, 2H), 2.94 (t, *J*=6.4 Hz, 2H). HRESIMS calcd for C_15_H_15_BrNO_2_ (**4c**) *m/z* [M+H]^+^ 320.0286, 322.0270 found 320.0284, 322.0267.

*3-bromo-N-(4'-cyano-[1,1'-biphenyl]-4-yl)propanamide (NS-260)* ***(4d)***: Light yellow powder containing *N*-(4'-cyano-[1,1'-biphenyl]-4-yl)acrylamide (**5d**) in the ratio of 1:1 (84%): ^1^H NMR (400 MHz, CDCl_3_) δ: (**4d**): 7.73-7.65 (m, 8H), 7.32 (br s, 1H), 3.74 (t, *J*=6.4 Hz, 2H), 2.99 (t, *J*=6.4 Hz, 2H). (**5d**): 7.73-7.65 (m, 8H), 6.48 (dd, *J*=16.8, 1.2 Hz, 1H), 6.27 (dd, *J*=16.8, 10.4 Hz, 1H), 5.83 (dd, *J*=10.4, 1.2 Hz, 1H). HRESIMS calcd for C_16_H_13_BrN_2_NaO (**4d**) *m/z* [M+Na]^+^ 351.0109, 353.0090 found 351.0108, 353.0087.

*N-([1,1'-biphenyl]-3-yl)-3-bromopropanamide (NS-264)* ***(4e)***: Pinkish powder (81%) containing about 50% of *N*-([1,1'-biphenyl]-3-yl)acrylamide (**5e**): ^1^H NMR (400 MHz, CDCl_3_) δ: (**4e**): 7.85 (s, 1H), 7.60-7.35 (m, 9H), 3.74 (t, *J*=6.4 Hz, 2H), 2.97 (t, *J*=6.4 Hz, 2H). (**5e**): 7.79 (s, 1H), 7.60-7.35 (m, 8H), 6.47 (dd, *J*=16.8, 1.2 Hz, 1H), 6.27 (dd, *J*=16.8, 10.4 Hz, 1H), 5.80 (dd, *J*=10.0, 1.2 Hz, 1H). HRESIMS calcd for C_15_H_14_BrNNaO (**4e**) *m/z* [M+Na]^+^ 326.0156, 328.0143 found 326.0152, 328.0138.

*N-(3-(benzyloxy)phenyl)-3-bromopropanamide (NS-261)* ***(4f)***: White powder (73%) containing *N*-(3-(benzyloxy)phenyl)acrylamide (**5f**) in the ratio of 1:1.6 : ^1^H NMR (400 MHz, CDCl_3_) δ: (**4f**): 7.49-7.00 (m, 9H), 6.75 (d, *J*=8.4 Hz, 1H), 5.07 (s, 2H), 3.71 (t, *J*=6.4 Hz, 2H), 2.94 (t, *J*=6.4 Hz, 2H). (**5f**): 7.49-7.00 (m, 8H), 6.75 (d, *J*=8.4 Hz, 1H), 6.44 (dd, *J*=16.8, 1.6 Hz, 1H), 6.23 (dd, *J*=17.2, 10.4 Hz, 1H), 5.78 (dd, *J*=10.4, 1.2 Hz, 1H), 5.08 (s, 2H). HRESIMS calcd for C_16_H_16_BrNNaO_2_ (**4f**) *m/z* [M+Na]^+^ 356.0262, 358.0241 found 356.0261, 358.0239.

*3-bromo-N-(5-(4-fluorophenyl)isoxazol-3-yl)propanamide (NS-274)* ***(4g)***: White powder (68%) containing about 50% of *N*-(5-(4-fluorophenyl)isoxazol-3-yl)acrylamide (**5g**): ^1^H NMR (400 MHz, CDCl_3_) δ: (**4g**): 8.64 (br s, 1H), 7.83-7.78 (m, 2H), 7.26 (s, 1H), 7.19 (t, *J*=8.4 Hz, 2H), 3.72 (t, *J*=6.4 Hz, 2H), 3.07 (t, *J*=6.4 Hz, 2H). (**5g**): 8.77 (br s, 1H), 7.83-7.78 (m, 2H), 7.31 (s, 1H), 7.19 (t, *J*=8.4 Hz, 2H), 6.53 (dd, *J*=16.8, 1.2 Hz, 1H), 6.33 (dd, *J*=16.8, 10.0 Hz, 1H), 6.92 (dd, *J*=10.4, 1.2 Hz, 1H). HRESIMS calcd for C_12_H_10_BrFN_2_NaO_2_ (**4g**) *m/z* [M+Na]^+^ 334.9807, 336.9795 found 334.9801, 336.9790.

*3-bromo-N-(3-(4-chlorophenyl)isoxazol-5-yl)propanamide (NS-273)* ***(4h)***: Off white crystals (65%), containing *N*-(3-(4-chlorophenyl)isoxazol-5-yl)acrylamide (**5h**) in the ratio of 1.4:1 : ^1^H NMR (400 MHz, CDCl_3_) δ: (**4h**): 8.13 (br s, 1H), 7.75 (d, *J*=8.4 Hz, 2H), 7.44 (d, *J*=8.8 Hz, 2H), 6.74 (s, 1H), 3.71 (t, *J*=6.4 Hz, 2H), 3.05 (t, *J*=6.4 Hz, 2H). (**5h**): 8.16 (br s, 1H), 7.76 (d, *J*=8.4 Hz, 2H), 7.44 (d, *J*=8.8 Hz, 2H), 6.78 (s, 1H), 6.55 (d, *J*=16.8, 1H), 6.28 (dd, *J*=16.8, 10.8 Hz, 1H), 5.95 (d, *J*=10.8 Hz, 1H). HRESIMS calcd for C_12_H_11_BrClN_2_O_2_ (**4h**) *m/z* [M+H]^+^ 328.9692, 330.9670 found 328.9693, 330.9671.

**References:**

1. Lasalle, M.; Picon, S.; Boulahjar, R.; Hoguet, V.; Van Obbergen, J.; Roussel, P.; Deprez, B.; Charton, J. Access to newly functionalized imidazole derivatives: efficient synthesis of novel 5-​amino-​2-​thioimidazoles using propylphosphonic anhydride (®T3P). *Tetrahedron Letters* (**2015**), 56(8), 1011-1014.
2. Sionogi and Co., Ltd., Japan. Antimycotic formulations containing isoxazoles. Jpn. Kokai Tokkyo Koho (**1984**). JP59128326 A 19840724 (Japanese).
3. Chemical library, no references.
4. Inui N, Nagasaki H, Yachigo S, Oikawa M,. Acrylamide derivatives as grip enhancers for tires. Eur. Pat. Appl. (1991). EP 409565 A1 Jan. 23, 1991, English.
5. Commercially available, no references.

**REPRESENTATIVE SPECTRA**


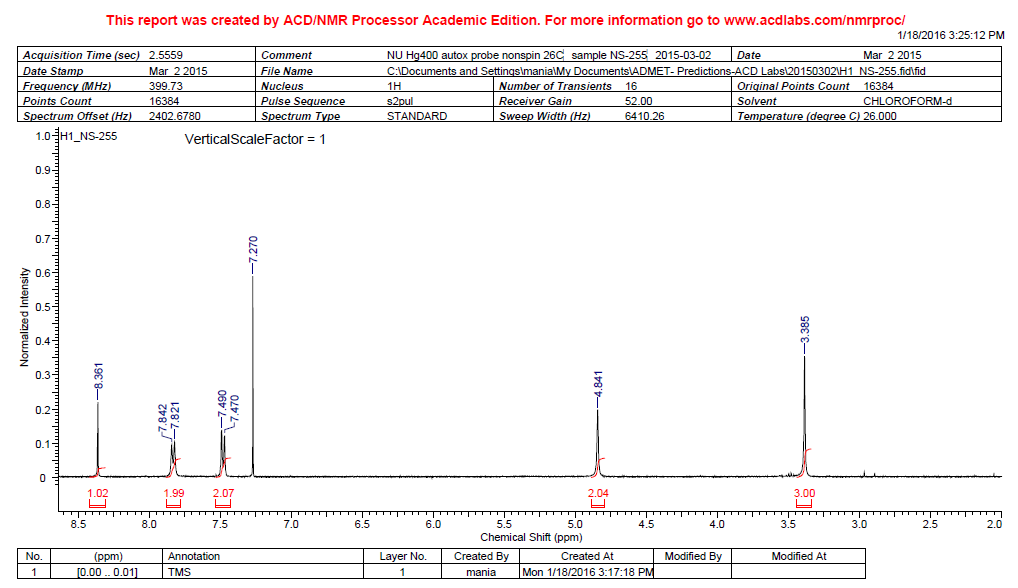


^1^H NMR spectrum of compound **2**


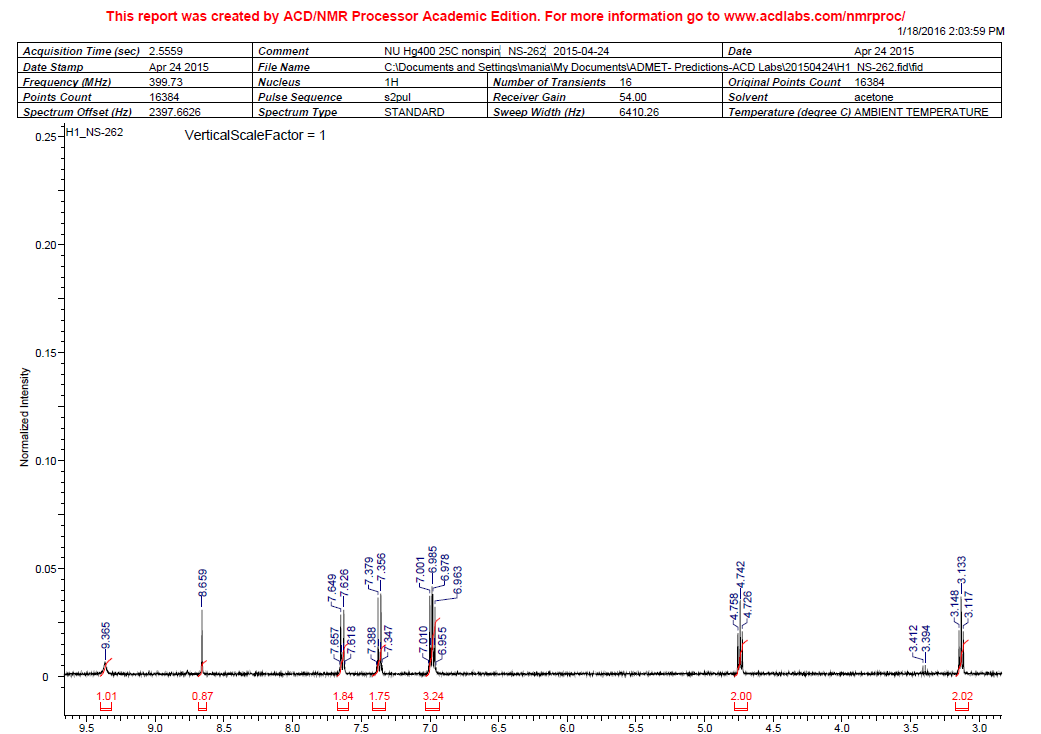


^1^H NMR spectrum of compound **6**


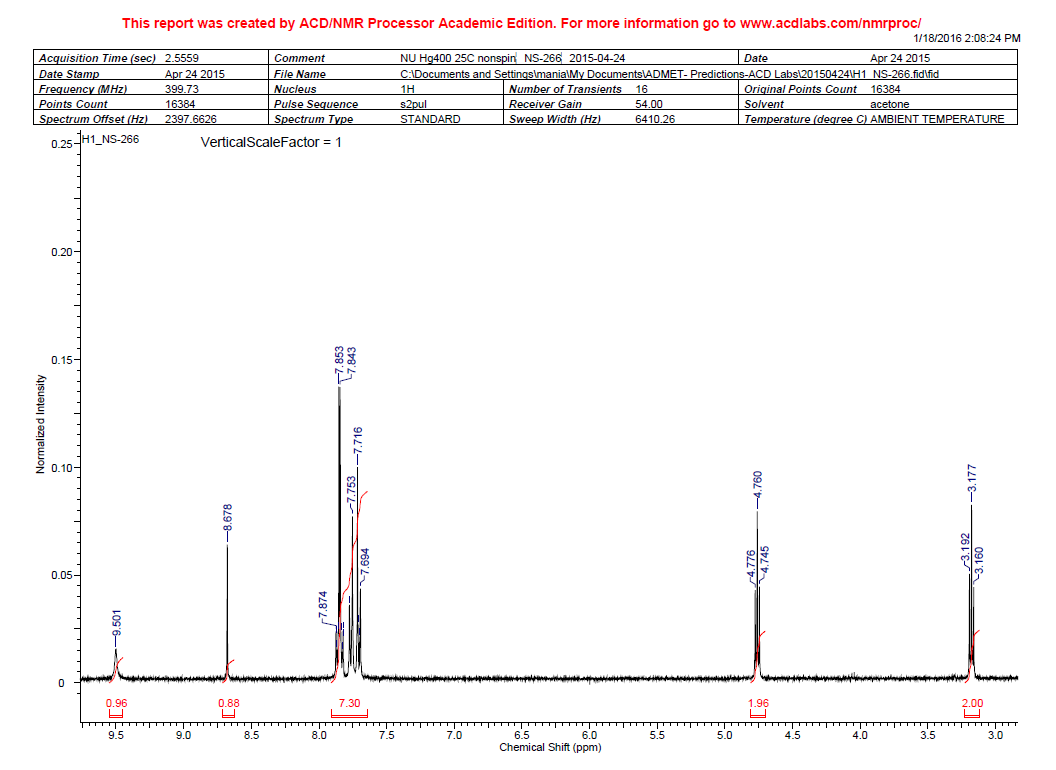


^1^H NMR spectrum of compound **9**


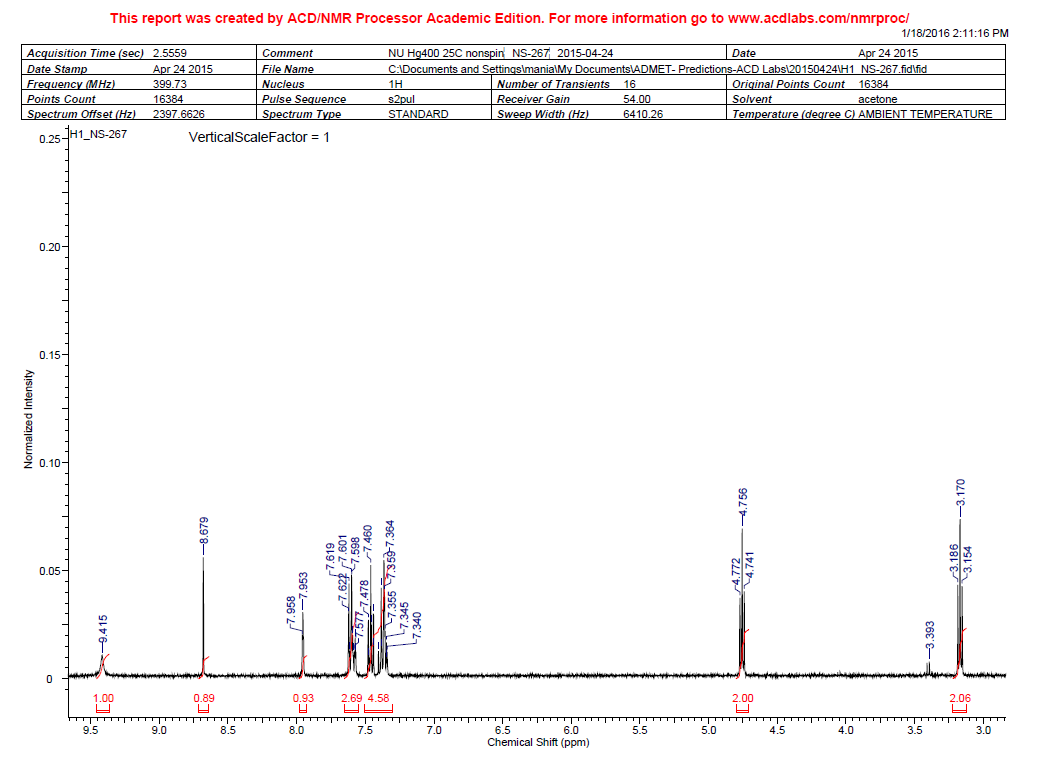


^1^H NMR spectrum of compound **10**


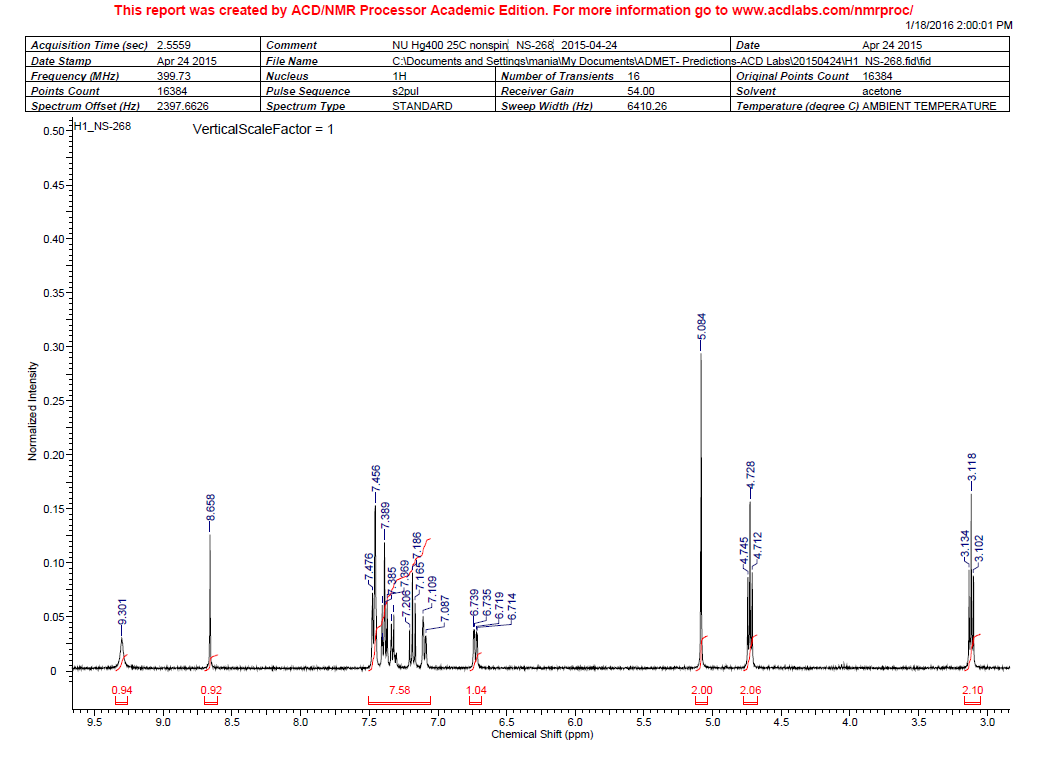


^1^H NMR spectrum of compound **11**


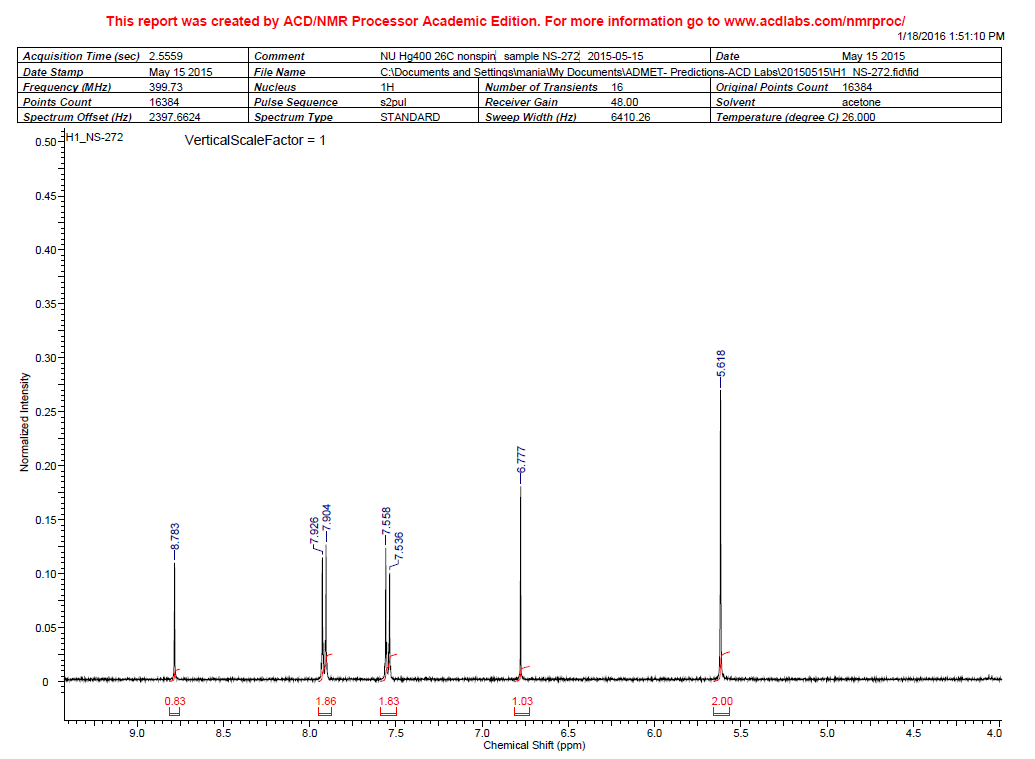


^1^H NMR spectrum of compound **15**


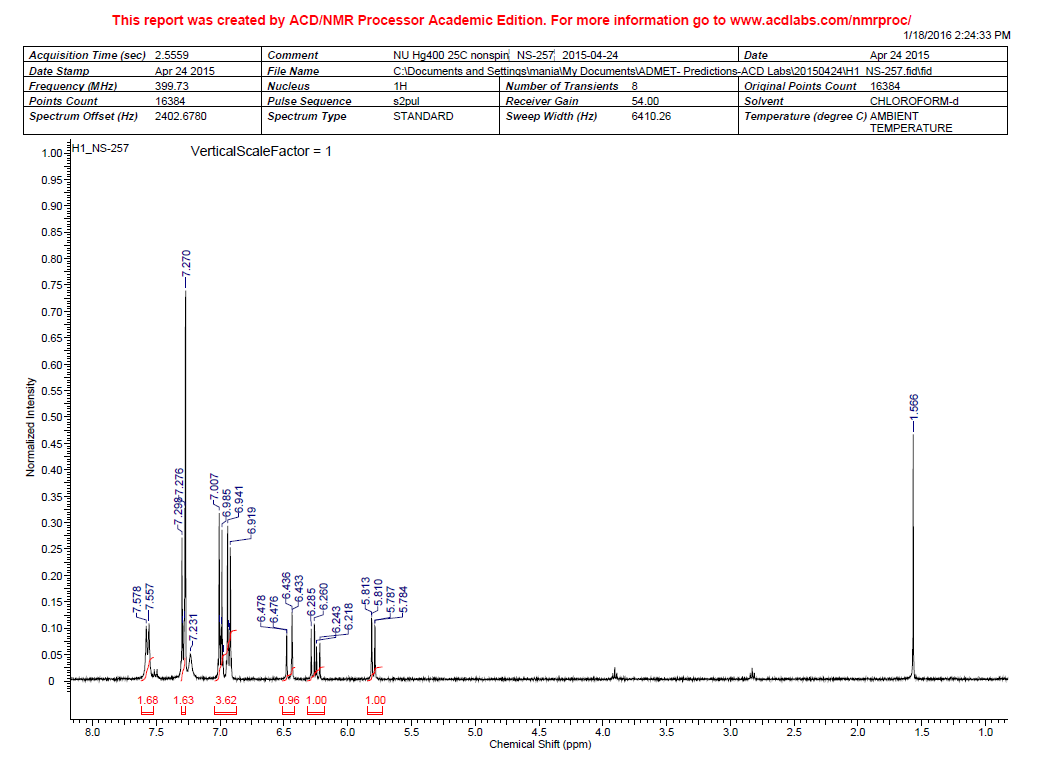


^1^H NMR spectrum of compound **5a**


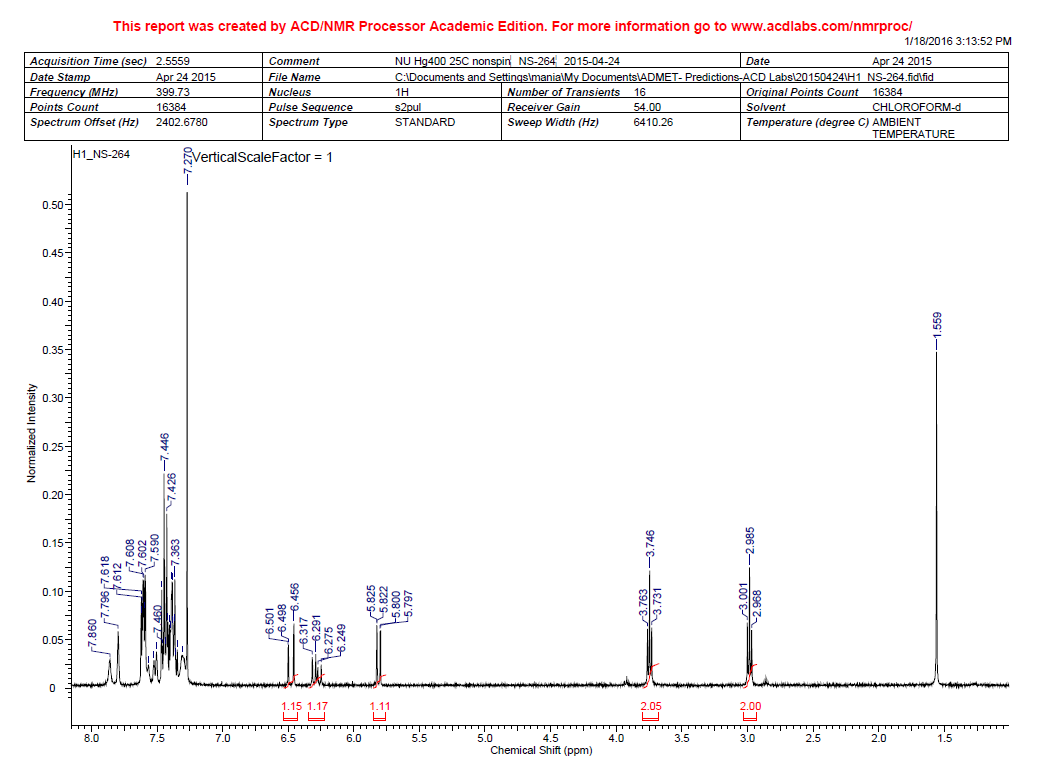


^1^H NMR spectrum of compounds **4e** and **5e** as mixture.


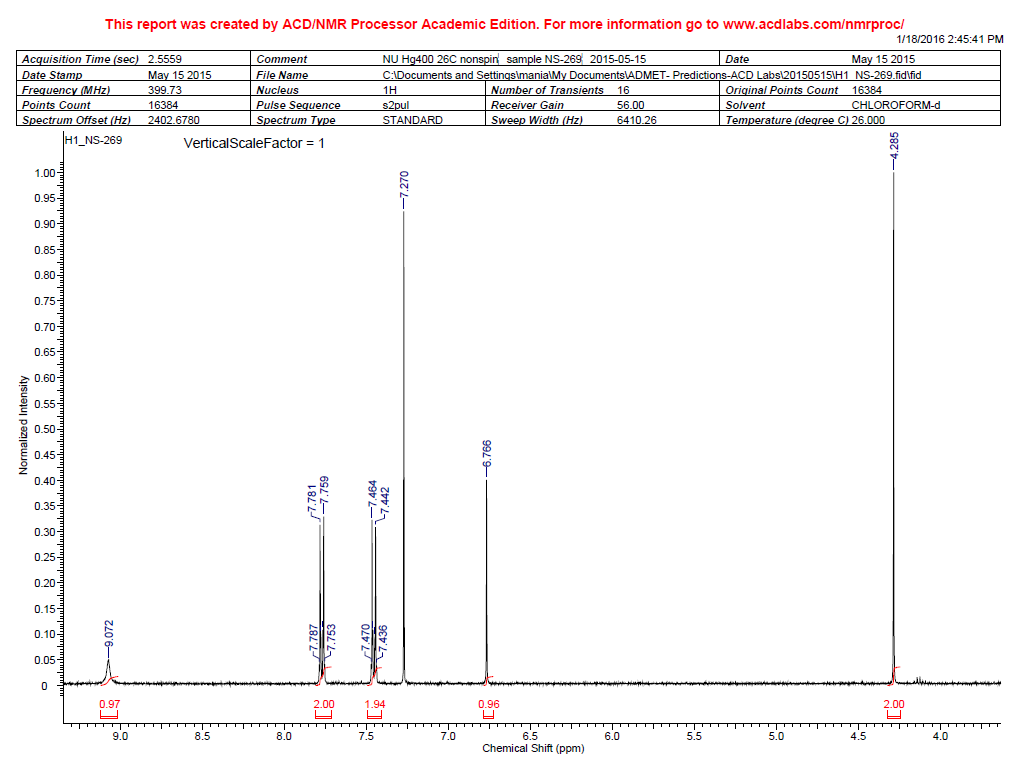


^1^H NMR spectrum of compounds **1c**.


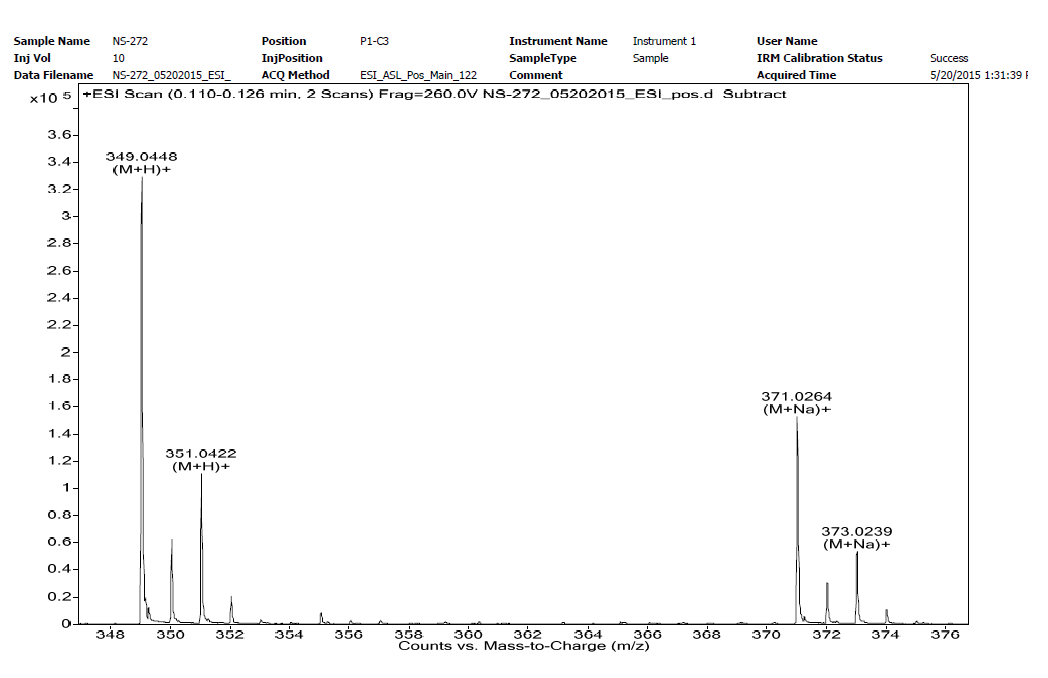


HRMS of compound **15**


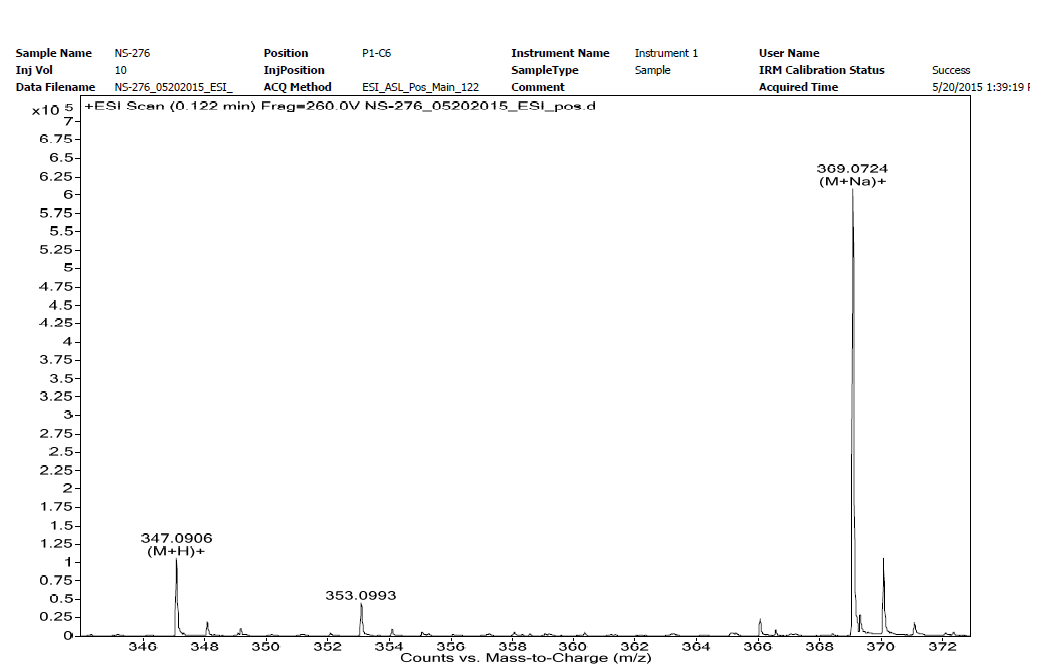


HRMS of compound **12**


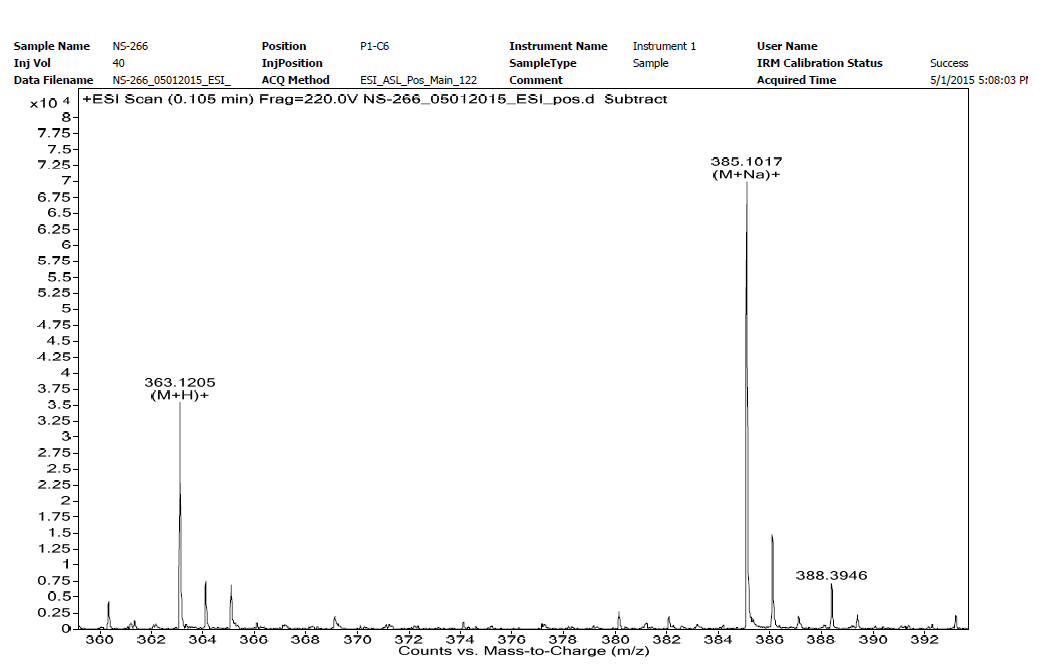


HRMS of compound **9**.
